# Psychosomatic relationships between the state of mental health and the level of vital threat of dermatological disease

**DOI:** 10.1192/j.eurpsy.2024.761

**Published:** 2024-08-27

**Authors:** M. Markova, T. Abdriakhimova, H. Skrebtsova, M. Chemerys

**Affiliations:** ^1^Kharkiv National Medical University, Kharkiv; ^2^Bogomolets National Medical University, Kyiv; ^3^Danylo Halytsky Lviv National Medical University, Lviv, Ukraine

## Abstract

**Introduction:**

According to the literature, 25-60% of dermatological patients have mental disorders. In the case of oncodermatological disease, the patient is under the influence of two stressogenic factors – existential experiences and social discomfort from the manifestations of the disease, which imprints on the patient’s mental health and promotes the development of mental maladaptation (MM).

**Objectives:**

To study the features of mental state in patients with dermatological diseases with different levels of vital threat.

**Methods:**

The examination included the use of clinical-psychological, psychodiagnostic and psychometric research methods.

**Results:**

120 dermatological patients were examined: 60 patients with non-vital dermatological diseases (L82, A63.0, D18.0, L80), and 60 patients with dermatological diseases posing a vital threat (C43, C44, D04).

The identification of clinical signs of MM proved their presence in 70 (58.4%) people in the total sample. Among patients with non-vital diseases, the signs of MM were established in 33 (55.0%), among the patients with vital diseases – in 37 (61.7%). So, among patients with dermatological diseases, there are both psychologically adapted and maladapted individuals, regardless of the vitality/non-vitality of the pathological process.

In dermatological patients with signs of MM, the clinical picture is dominated by anxious (mainly in patients with non-vital diseases) and depressive (mainly in patients with vital diseases) radicals. Auxiliary psychopathological constructs are represented by manifestations of somatization, obsessive-compulsive symptoms, interpersonal sensitivity, and phobic anxiety. Affective symptoms are most pronounced in patients with MM and vital diseases, it is less pronounced in patients with non-vital pathology.

The presence and intensity of maladaptive pathopsychological-affective reactions in patients with dermatological pathology are not clearly associated with the vitality of the dermatological process, but are based on mechanisms of the mutual influence of biological predisposition and psychological and psychosocial factors, the mosaic combination of which determines the individual’s resource capabilities for constructive acceptance the fact of the presence of a dermatological disease and the development of an adequate strategy for its mastery, regardless of the severity of the disease.

**Conclusions:**

These patterns should be considered when developing treatment measures and rehabilitation for patients with dermatological pathology.

**Disclosure of Interest:**

None Declared

